# Theoretical Model of Helium Bubble Growth and Density in Plasma-Facing Metals

**DOI:** 10.1038/s41598-020-58581-8

**Published:** 2020-02-10

**Authors:** Karl D. Hammond, Dimitrios Maroudas, Brian D. Wirth

**Affiliations:** 1 Department of Biomedical, Biological, and Chemical Engineering, University of Missouri, Columbia, MO 65211 USA; 2 Nuclear Engineering Program, University of Missouri, Columbia, MO 65211 USA; 3 Department of Chemical Engineering, University of Massachusetts, Amherst, MA 01003 USA; 4 Department of Nuclear Engineering, University of Tennessee, Knoxville, TN 37996 USA; 5 Fusion Energy Division, Oak Ridge National Laboratory, Oak Ridge, TN 37831 USA

**Keywords:** Nuclear fusion and fission, Theory and computation, Metals and alloys, Thermodynamics, Applied physics

## Abstract

We present a theoretically-motivated model of helium bubble density as a function of volume for high-pressure helium bubbles in plasma-facing tungsten. The model is a good match to the empirical correlation we published previously [Hammond *et al*., *Acta Mater*. **144**, 561–578 (2018)] for small bubbles, but the current model uses no adjustable parameters. The model is likely applicable to significantly larger bubbles than the ones examined here, and its assumptions can be extended trivially to other metals and gases. We expect the model to be broadly applicable and useful in coarse-grained models of gas transport in metals.

## Introduction

Helium bubble growth in plasma-facing materials (PFMs) is an important aspect of nuclear fusion materials research^1–3^. Not only do helium bubbles cause material embrittlement and fatigue, but helium also causes significant damage to plasma-facing surfaces in the form of fuzz or coral-like features^4–7^, and modelling these phenomena continues to be a challenge^8–11^. Experimental evidence indicates that helium bubbles on the order of one nanometre in diameter are present in at least the first 20–30 nm of tungsten beneath its plasma-exposed surface at concentrations high enough to observe with transmission electron microscopy^12^, and a study by Yi *et al*.^13^ of bubbles formed by high-temperature, high-ion-energy injection of helium suggested that large bubbles have a He/V ratio in the range 2–5, depending on the temperature. Helium bubbles also form in radioactive materials and irradiated materials through *α*-decay processes, though the rates of bubble formation and growth in such systems are typically very slow compared to the processes at work in plasma-facing materials. Efforts to model helium bubble growth are consequently relatively common in the fission community^14–17^.

We previously used molecular dynamics (MD) simulations to study tungsten that was exposed to 100 eV helium plasma at a temperature of 933 K over a range of fluxes and surface orientations for times on the order of microseconds^18–20^. In one of these studies^19^, we suggested a two-parameter empirical correlation for the number of helium atoms, *n*_He_, in a helium bubble as a function of the number of vacancies, *n*_*V*_, generated during the formation of the bubble—equivalent to the density (helium-to-vacancy ratio) as a function of bubble size—namely,

1 $${n}_{{\rm{H}}{\rm{e}}}=5{n}_{{\rm{V}}}^{0.86}.$$

This correlation, much like other studies of helium bubble energetics and bubble growth in tungsten^21–30^, has been useful in developing coarse-grained models of helium transport and surface morphological evolution in tungsten^31–36^. However, the correlation in Eq. 1 has no particular theoretical foundation. Such a theoretical foundation is required to make this type of density–volume relation transferable over a broad range of conditions and, accordingly, PFM thermodynamic states. We present here theoretical equations for bubble density as a function of size based on simple physical models and equations of state. These equations provide theoretical support to a massive amount of data generated from the analysis of large-scale MD simulations of tungsten surface evolution associated with helium implantation^18–20^. These MD simulations span several orders of magnitude in helium flux (Γ ~ 10^25^–10^28^ m^−2^s^−1^) and fluence (up to Φ ~ 10^21^ m^−2^) and have achieved both the lowest helium fluxes and highest helium fluences through direct atomistic simulation yet reported in the literature^20^, thereby rendering these simulations more representative of experiments in plasma devices than other MD simulations published to date: although the fluence is still low compared to typical experiments, the lowest flux is only one order of magnitude higher than what is expected in ITER^37^. The data science methods involved in the analysis of the enormous data sets generated from the MD simulations have been presented and discussed in our prior work^18–20^.

The pressure in a helium bubble that is far from a surface or grain boundary and which has not yet burst should be greater than or equal to the equilibrium pressure, *P*_eq_. As established in prior work^38–44^, pressure in growing bubbles is relieved by “punching out” dislocation loops, called “loop-punching”. The pressure should therefore be less than or approximately equal to the loop-punching stress; as helium is arriving at the bubbles much faster than vacancies are, we expect the pressure inside most bubbles to be closer to the critical value corresponding to loop-punching than to the equilibrium pressure.

Assuming spherical bubbles, the equilibrium pressure increase across a metal–gas interface in a bubble of gas is given by the Young–Laplace equation,

2 $$\Delta {P}_{{\rm{eq}}}=\frac{2\gamma }{{r}_{B}},$$

where *r*_*B*_ is the radius of the bubble and *γ* is the bubble surface tension, that is, the interfacial tension at the helium/metal interface. We will assume that the pressure in the metal itself—which is exposed to a free surface—is negligible, meaning that Δ*P*_eq_ is equal to the pressure in the bubble at equilibrium. The Young–Laplace equation applies in the continuum limit, meaning it is applicable to bubbles larger than a particular size for which the discrete nature of the atoms making up the interface can be ignored. It has been suggested that this limit corresponds to a radius of 5–8 molecular diameters for the menisci of wetting interfaces^45,46^ and as small as 1 molecular diameter for non-wetting interfaces^47^; the non-wetting situation is applicable to metal–helium interfaces.

The additional pressure required to expand a spherical bubble by loop-punching is strongly coupled to the volume before and after the loop punches, and there is presumably some elastic rebound after the loop forms. This correspondence for a spherical bubble was estimated by Wolfer^48^ to be

3 $$V={V}_{0}+{V}_{L}+\pi {r}_{B}^{3}(\frac{P-2\gamma /{r}_{B}}{G}-\frac{b{r}_{L}^{2}}{{R}^{2}}),$$

where *V* is the volume of the bubble after loop-punching, *V*_0_ is the volume of the bubble prior to loop-punching, *V*_*L*_ is the volume taken up by the loop prior to loop-punching (such that ${V}_{0}+{V}_{L}=\frac{4}{3}\pi {r}_{B}^{3}$), *r*_*L*_ is the radius of the prismatic loop, *G* is the shear modulus, *b* is the magnitude of the dislocation loop’s Burgers vector, and *R* is the distance from the center of the bubble to the loop periphery. At the instant the loop punches out, *R* ≈ *r*_*B*_ and *r*_*L*_ ≈ *r*_*B*_. If we assume the final volume (post–loop-punching) of the bubble is proportional to the initial volume by the parameter *ω* (i.e., that $V=\omega ({V}_{0}+{V}_{L})=\frac{4}{3}\pi \omega {r}_{B}^{3}$), then Eq. 3 with these approximations can be rearranged to

4 $$P=\frac{2\gamma +Gb}{{r}_{B}}+\frac{4G}{3}(\omega -1),$$

where 0 < *ω* ≤ 1 are logical values: elastic relaxation will tend to make the bubble *smaller*, not larger, once the loop punches out. A value of *ω* = 1, which must hold in the limit of large bubbles, yields *P* = 2*γ*/*r* + *Gb*/*r*, which is consistent with earlier work by Trinkaus and Wolfer^49,50^. The bubble pressure should therefore be bounded, approximately, by the inequalities

5 $$\frac{2\gamma }{{r}_{B}}\le P < \frac{2\gamma +Gb}{{r}_{B}}$$

for spherical bubbles. Because the rate of arrival of helium at most bubbles far exceeds the rate of arrival of vacancies or interstitials, except in cases when punched-out loops detach from one bubble and annihilate inside another, we expect the pressure in helium bubbles to be closer to the loop-punching pressure than the equilibrium pressure. Near surfaces, the loop-punching stress is lower than in the bulk^51^, meaning the pressure in those bubbles would still lie in this range but would be closer to the lower bound than the upper bound.

It should be noted that the pressure inside a bubble is above the loop-punching threshold just prior to punching out a loop, and it drops below the threshold after the loop forms^49^. With this in mind, we expect that a correlation based on the loop-punching threshold would be a good indicator of the average bubble size observed in molecular dynamics simulations. In situations in which the helium implantation flux is sufficiently low or in which the helium ion energy is sufficient to produce Frenkel pairs, the rate of helium arrival at the bubble would be comparable to the rate of vacancy transport and/or creation. In such situations, the density would drop to something closer to the equilibrium value.

The volume of a spherical bubble is approximately $V={n}_{{\rm{V}}}\Omega =4\pi {r}_{B}^{3}/3$, where Ω is the atomic volume of the metal. The bubble radius, *r*_*B*_, is therefore ${r}_{B}={(3{n}_{{\rm{V}}}\Omega /4\pi )}^{1/3}$. The pressure in the bubble should therefore satisfy the inequalities

6 $$\frac{2\gamma }{{\left(\frac{3}{4\pi }{n}_{{\rm{V}}}\Omega \right)}^{1/3}}\le P < \frac{2\gamma +Gb}{{\left(\frac{3}{4\pi }{n}_{{\rm{V}}}\Omega \right)}^{1/3}}.$$

For a correlation of *n*_He_ as a function of *n*_V_, we need a relationship between *P* and *n*_V_ and/or *n*_He_; that is, we need an equation of state. We will discuss expressions for *n*_He_ as a function of *n*_V_ that are consistent with four equations of state for helium: the ideal gas law, the virial equation truncated after the second term in both pressure- and volume-explicit forms, and the empirical equation of Benedict^52^ as parametrised for helium by Mills, Liebenberg, and Bronson^53^.

## Results and Discussion

### Bubbles of ideal gas

If the gas inside the bubble obeys the ideal gas law—that is, *Pn*_V_Ω = *n*_He_*kT*, where *k* is the Boltzmann constant and *T* is the absolute temperature—then the inequalities in Eq. 6 become

7 $$\frac{2\gamma }{{\left(\frac{3}{4\pi }{n}_{{\rm{V}}}\Omega \right)}^{1/3}}\le \frac{{n}_{{\rm{He}}}kT}{{n}_{{\rm{V}}}\Omega } < \frac{2\gamma +Gb}{{\left(\frac{3}{4\pi }{n}_{{\rm{V}}}\Omega \right)}^{1/3}}.$$

Solving for *n*_He_ gives the inequalities

8 $$\frac{2\gamma \,{\Omega }^{2/3}}{{(3/4\pi )}^{1/3}kT}{n}_{{\rm{V}}}^{2/3}\le {n}_{{\rm{H}}{\rm{e}}} < \frac{(2\gamma +Gb){\Omega }^{2/3}}{{(3/4\pi )}^{1/3}kT}{n}_{{\rm{V}}}^{2/3}.$$

This correlation is extremely inaccurate, as we shall see later. However, it suggests that an equation of the form

9 $${n}_{{\rm{He}}}\propto \frac{{n}_{{\rm{V}}}^{2/3}}{T}$$

might be a reasonable empirical model. A similar model was proposed by Suzudo *et al*.^54^ for helium in metals based on a lattice model.

Equation 8 assumes spherical bubbles, but many of the bubbles observed in our database of relatively large simulations^18–20^ are oblate. Some of the bubbles near the surface are almost cylindrical, owing to the ease of emitting dislocation loops that rapidly relieve stress by piling up on the surface (see Sefta *et al*.^38,51^). Non-spherical bubbles are more likely to occur when bubbles are near the surface and the rate of helium addition is slow relative to the rate of interstitial diffusion^55^. If we assume the bubbles are cylindrical, then

10 $$V=\pi {r}^{2}h={n}_{{\rm{V}}}\Omega ,$$

where *h* is the length of the cylinder in the axial direction and *r* is the radius of the cylinder. The bubble pressure condition of Eq. 5 becomes

11 $$\frac{\gamma }{r}\le P < \frac{\gamma +Gb}{r},$$

where the Young–Laplace equation has been rewritten for cylindrical bubbles.

Previous simulations of helium bubble growth in tungsten^38–40,56^ indicate that the dislocation loops that are emitted have approximately the same radius, and in cases in which elongated bubbles are observed, those bubbles grow in the axial direction with relatively constant radius. It is not entirely clear whether this will persist when the bubbles generating the prismatic loops grow larger and/or deeper beneath the surface, but if we assume every bubble has a similar diameter (i.e., *r* = *R*_cyl_*a*_0_ = *R*_cyl_(2Ω)^1/3^ for body-centred cubic materials such as tungsten, where *R*_cyl_ is a constant), we can eliminate *r* in favour of *R*_cyl_ and Ω, which yields

12 $$\frac{\gamma \,{\Omega }^{2/3}{n}_{{\rm{V}}}}{{2}^{1/3}{R}_{{\rm{c}}{\rm{y}}{\rm{l}}}kT}\le {n}_{{\rm{H}}{\rm{e}}} < \frac{(\gamma +Gb){\Omega }^{2/3}{n}_{{\rm{V}}}}{{2}^{1/3}{R}_{{\rm{c}}{\rm{y}}{\rm{l}}}kT}.$$

This suggests a correlation of the form

13 $${n}_{{\rm{He}}}\propto \frac{{n}_{{\rm{V}}}}{T}.$$

Equations 9 and 13 indicate that an equation of the form

14 $${n}_{{\rm{He}}}=C{n}_{{\rm{V}}}^{\alpha },$$

where α ∈ [2/3, 1) and the parameter *C* has an inverse temperature dependence, is reasonable. This is consistent with the correlation posited in our prior work^19^, which is reproduced in Eq. 1. However, the theoretically-derived parameters are much larger than would be required for a good fit, suggesting that the ideal gas law is (unsurprisingly) not good enough for this particular application. It should also be noted that the assumption that *R*_cyl_ is a universal constant is likely inaccurate, particularly for large bubbles; therefore, *R*_cyl_ should in general be viewed as a dimensionless parameter over a representative range of numerical values.

### Bubbles obeying the pressure-explicit virial equation of state

Toward the goal of making this correlation predictive, let us consider bubbles whose contents obey the pressure-explicit virial equation of state^57^,

15 $$P=\frac{{n}_{{\rm{H}}{\rm{e}}}kT}{V}+\frac{{n}_{{\rm{H}}{\rm{e}}}^{2}BkT}{{V}^{2}}+\frac{{n}_{{\rm{H}}{\rm{e}}}^{3}CkT}{{V}^{3}}+\cdots .$$

If we ignore terms beyond the second term (i.e., *C* = *D* = … = 0), then for spherical bubbles,

16 $$\frac{2\gamma }{{(\frac{3}{4\pi })}^{1/3}{n}_{{\rm{V}}}^{1/3}{\Omega }_{}^{1/3}}\le P=\frac{{n}_{{\rm{H}}{\rm{e}}}kT}{{n}_{{\rm{V}}}\Omega }+B\frac{{n}_{{\rm{H}}{\rm{e}}}^{2}kT}{{n}_{{\rm{V}}}^{2}{\Omega }^{2}} < \frac{2\gamma +Gb}{{(\frac{3}{4\pi })}^{1/3}{n}_{{\rm{V}}}^{1/3}{\Omega }_{}^{1/3}}.$$

The neglect of the term containing *C* and all higher-order terms is motivated partially by convenience—the resulting correlation with *C* ≠ 0 would be a more complicated function to express analytically—and partially from the fact that high-order virial coefficients for helium at temperatures relevant to fusion reactor materials (≈1000 K) are not generally available.

The algebraic equation for *n*_He_ in Eq. 16 is quadratic, and yields the physical solution

17 $$\frac{{n}_{{\rm{V}}}\Omega }{2B}[-\,1+\sqrt{1+\frac{8\gamma \,B}{kT{F}_{s}{n}_{{\rm{V}}}^{1/3}}}]\le {n}_{{\rm{H}}{\rm{e}}} < \frac{{n}_{{\rm{V}}}\Omega }{2B}[-\,1+\sqrt{1+\frac{4B(2\gamma +Gb)}{kT{F}_{s}{n}_{{\rm{V}}}^{1/3}}}],$$

where we have consolidated the geometric parameter *F*_*s*_ = (3Ω/4*π*)^1/3^ for spheres. Similarly, a cylindrical bubble geometry with the truncated virial equation yields

18 $$\frac{{n}_{{\rm{V}}}\Omega }{2B}[-\,1+\sqrt{1+\frac{4\gamma \,B}{kT{F}_{c}}}]\le {n}_{{\rm{H}}{\rm{e}}} < \frac{{n}_{{\rm{V}}}\Omega }{2B}[-\,1+\sqrt{1+\frac{4B(\gamma +Gb)}{kT{F}_{c}}}],$$

where *F*_*c*_ = *r* = (2Ω)^1/3^*R*_cyl_. Equation 18 is the same as Eq. 17 with the factor *F*_*s*_ replaced by ${F}_{c}{n}_{{\rm{V}}}^{-1/3}$ and *γ* replaced by *γ*/2. The same is true of Eqs. 8 and 12: replace *F*_*s*_ with ${F}_{c}{n}_{{\rm{V}}}^{-1/3}$ and *γ* with *γ*/2 to convert the spherical form to the cylindrical form. With this observation in mind, we will only give explicit formulas for the spherical forms from this point onward.

### Bubbles obeying the volume-explicit virial equation of state

The virial equation of state, an infinite series, can also be written in volume-explicit form^57^, viz.,

19 $$V=\frac{{n}_{{\rm{H}}{\rm{e}}}kT}{P}(1+B\text{'}P+C\text{'}{P}^{2}+\cdots ).$$

In the infinite series limit, the two forms are equivalent if ${B}^{^{\prime} }=B/kT$, ${C}^{^{\prime} }=(C-{B}^{2})/{(kT)}^{2}$, and so forth. Truncating the volume-explicit form after the second term yields

20 $$V={n}_{{\rm{V}}}\Omega =\frac{{n}_{{\rm{H}}{\rm{e}}}kT}{P}+{n}_{{\rm{H}}{\rm{e}}}B.$$

Combining this expression with the inequalities in Eq. 6 results in the inequalities

21 $$\frac{2\gamma \,{n}_{{\rm{V}}}\Omega }{2\gamma \,B+kT{n}_{{\rm{V}}}^{1/3}{F}_{s}}\le {n}^{{\rm{H}}{\rm{e}}} < \frac{(2\gamma +Gb){n}_{{\rm{V}}}\Omega }{(2\gamma +Gb)B+kT{n}_{{\rm{V}}}^{1/3}{F}_{s}}$$

Again, equivalent expressions for cylindrical bubbles can be obtained by replacing *γ* with *γ*/2 and *F*_*s*_ by ${F}_{c}{n}_{{\rm{V}}}^{-1/3}$.

**Figure 1:**
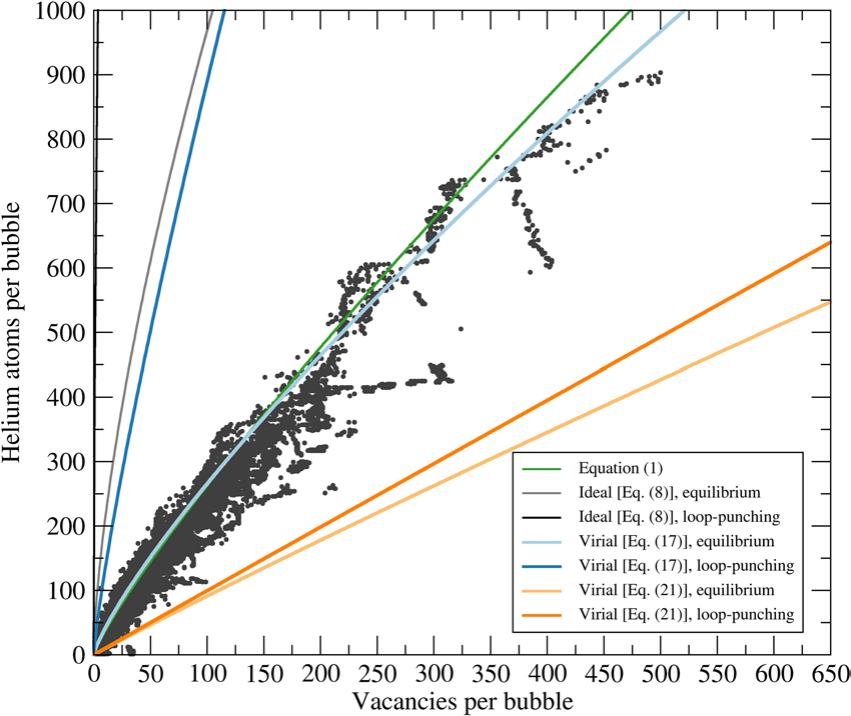
Plots of the original correlation (Eq. 1) and the spherical bubble models for the ideal gas (Eq. 8) and virial (Eqs. 17 and 21) equations of state. Points correspond to bubbles at any given instant in time at depths greater than 5 nm as collected from seven molecular dynamics simulations spanning several orders of magnitude in flux and fluence with different system sizes and surface orientations^19,20^.

We show plots of the correlations developed from the ideal gas and virial equations of state (both pressure- and volume-explicit varieties) with parameters from the literature (listed in Table 1), along with bubble size and density data derived from a series of previously-published MD simulations^18–20^, in Fig. 1. The MD-derived bubble sizes are expressed in terms of the number of vacancies eclipsed by the helium in the bubble, which in turn are determined by comparing the tungsten atoms’ positions in the system to a perfect lattice; a site is “vacant” if there are no tungsten atoms within 0.6 *a*_0_ of it. It should be noted that a particular bubble may have been created by punching out a different number of Frenkel pairs than are identified by this algorithm because of crystal relaxation effects, but that the volume, *V*, of a bubble as identified in this manner is always *V* ≈ *n*_V_Ω.

**Table 1:** Parameters and physical constants used in the models. ^a^This value was determined from the average of 28 MD simulations at *P* = 0 and *T* = 933 K containing spherical cavities of various sizes using the tungsten EAM potential used in our prior studies^58–60^. Values for specific planar surfaces range from 2.58 J/m^2^ to 3.30 J/m^2^ with this potential^19^. ^b^Determined from a molecular statics simulation using the same potential.

Parameter	Value	Ref.
*T*	933 K	^19^
*γ*	2.67 J/m^2^	(this work^a^)
*a*_0_	0.3180 nm	^19^
Ω	${a}_{0}^{3}/2=1.6079\times 1{0}^{-29}$ m^3^	(deduced from *a*_0_)
*G*	156.1 GPa	(this work)^b^
*b*	$\frac{{a}_{0}\sqrt{3}}{2}=$ 0.2755 nm	(deduced from *a*_0_)
*k*	1.380649 × 10^−23^ J K^−1^	^61^
*B* (at 933 K)	9.63 cm^3^/mol = 1.60 × 10^−29^ m^3^	^62,63^
*A*_−1,1_	− 5.5991 × 10^−27^ m^3^ K^1/2^ Pa^1/3^	^53^
*A*_0,1_	1.7400 × 10^−26^ m^3^ Pa^1/3^	^53^
*A*_2,1_	4.9833 × 10^−30^ m^3^ K^−1^ Pa^1/3^	^53^
*A*_0,2_	− 4.4658 × 10^−24^ m^3^ Pa^2/3^	^53^
*A*_2,2_	− 8.7825 × 10^−27^ m^3^ K^−1^ Pa^2/3^	^53^
*A*_0,3_	1.7595 × 10^−22^ J	^53^
*A*_2,3_	1.7608 × 10^−23^ J K^−1^	^53^
*A*_−1,3_	− 3.2615 × 10^−21^ J K^1/2^	^53^
*A*_−2,3_	3.1524 × 10^−20^ J K	^53^

As is clear from Fig. 1, the ideal gas law is clearly unfit for this particular task. The pressure-explicit virial equation at equilibrium appears to be an excellent fit, but this is deceptive: we expect, based on bubble growth considerations, that the pressure (and density) in the bubble should be close to the values associated with loop-punching, not with equilibrium. Comparing the pressure-explicit virial equation to the volume-explicit virial equation confirms that the virial equation truncated at the second coefficient is not sufficiently accurate at high pressure.

### Bubbles obeying the Benedict equation of state

Finally, let us consider the equation of state of Benedict^52^,

22a $$\begin{array}{c}\frac{V}{{n}_{{\rm{H}}{\rm{e}}}}={f}_{1}(T){P}^{-1/3}+{f}_{2}(T){P}^{-2/3}+{f}_{3}(T){P}^{-1},\end{array}$$

where

22b $${f}_{1}(T)={A}_{-1,1}{T}^{-1/2}+{A}_{0,1}+{A}_{2,1}T$$

22c $${f}_{2}(T)={A}_{0,2}+{A}_{2,2}T$$

22d $${f}_{3}(T)={A}_{-2,3}{T}^{-1}+{A}_{-1,3}{T}^{-1/2}+{A}_{0,3}+{A}_{2,3}T$$

**Figure 2:**
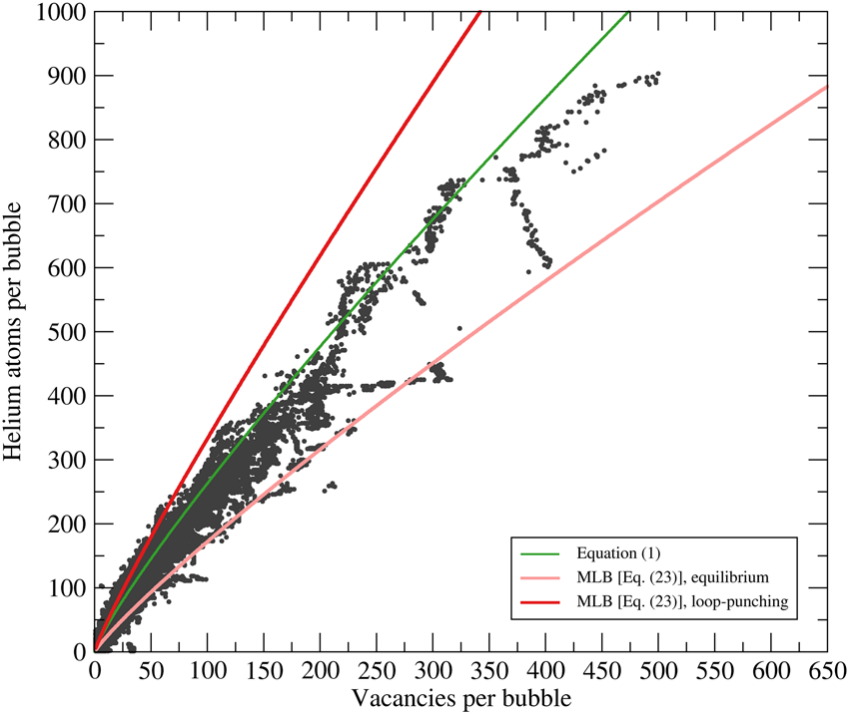
Results of the correlations based on the Benedict equation of state (Eq. 23), as well as the original correlation (Eq. 1). Points correspond to bubbles at any given instant in time at depths greater than 5 nm as collected from seven molecular dynamics simulations spanning several orders of magnitude in flux and fluence with different system sizes and surface orientations^19,20^.

**Figure 3:**
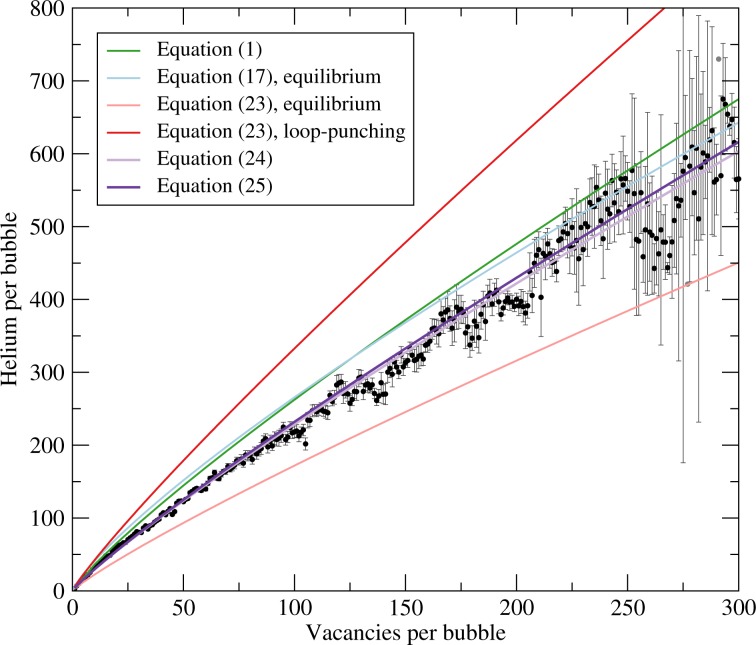
Performance of the empirical (Eq. 1) and theoretical models (Eqs. 17, 23, 24, and 25) against the average number of helium atoms in a bubble of a given size for helium atoms at distances greater than 4 nm beneath the original surface (i.e., the surface’s location prior to plasma exposure). All “averages” based on only one point are denoted in grey rather than black and lack error bars. Error bars denote 95% confidence intervals.

Parameters for Eq. 22 were determined for helium by Mills, Liebenberg, and Bronson^53^ based on a 670-point data set in the range 200 MPa < *P* < 2 GPa and 75 K < *T* < 300 K. We will refer to the Benedict equation with these parameters as the “MLB equation of state.” Substituting *P* from Eq. 6 and *V* = *n*_V_Ω in Eq. 22 gives the (rather complicated) expression

23 $$\begin{array}{c}\frac{{n}_{{\rm{V}}}\Omega }{{f}_{1}(T){F}_{s}^{1/3}{n}_{{\rm{V}}}^{1/9}{(2\gamma )}^{-1/3}+{f}_{2}(T){F}_{s}^{2/3}{n}_{{\rm{V}}}^{2/9}{(2\gamma )}^{-2/3}+{f}_{3}(T){F}_{s}{n}_{{\rm{V}}}^{1/3}{(2\gamma )}^{-1}}\le {n}_{{\rm{H}}{\rm{e}}}\\  < \frac{{n}_{{\rm{V}}}\Omega }{{f}_{1}(T){F}_{s}^{1/3}{n}_{{\rm{V}}}^{1/9}{(2\gamma +Gb)}^{-1/3}+{f}_{2}(T){F}_{s}^{2/3}{n}_{{\rm{V}}}^{2/9}{(2\gamma +Gb)}^{-2/3}+{f}_{3}(T){F}_{s}{n}_{{\rm{V}}}^{1/3}{(2\gamma +Gb)}^{-1}},\end{array}$$

where *F*_*s*_ = (3Ω/4*π*)^1/3^ is the same geometric factor as before. The performance of this model compared to the original correlation in Eq. 1 is shown in Fig. 2. We also show (Fig. 3) a plot corresponding to the geometric mean of the two inequalities in Eq. 23, which is

24 $$\begin{array}{ccc}{n}_{{\rm{H}}{\rm{e}}} & = & {n}_{{\rm{V}}}\Omega [{f}_{1}{(T)}^{2}{F}_{s}^{2/3}{(2\gamma )}^{-1/3}{(2\gamma +Gb)}^{-1/3}{n}_{{\rm{V}}}^{2/9}\\  &  & +{f}_{1}(T){f}_{2}(T){F}_{s}({(2\gamma )}^{-1/3}{(2\gamma +Gb)}^{-2/3}+{(2\gamma )}^{-2/3}{(2\gamma +Gb)}^{-1/3}){n}_{{\rm{V}}}^{1/3}\\  &  & +({f}_{1}(T){f}_{3}(T){(2\gamma )}^{-1/3}{(2\gamma +Gb)}^{-1}+{f}_{2}(T{)}^{2}{(2\gamma )}^{-2/3}{(2\gamma +Gb)}^{-2/3}\\  &  & +{f}_{1}(T){f}_{3}(T){(2\gamma )}^{-1}{(2\gamma +Gb)}^{-1/3}){F}_{s}^{4/3}{n}_{{\rm{V}}}^{4/9}\\  &  & +{f}_{2}(T){f}_{3}(T)({(2\gamma )}^{-1}{(2\gamma +Gb)}^{-2/3}+{(2\gamma )}^{-2/3}{(2\gamma +Gb)}^{-1}){F}_{s}^{5/3}{n}_{{\rm{V}}}^{5/9}\\  &  & +{f}_{3}(T{)}^{2}{F}_{s}^{2}{(2\gamma )}^{-1}{(2\gamma +Gb)}^{-1}{n}_{{\rm{V}}}^{2/3}{]}^{-1/2}.\end{array}$$

Again, one can convert to a cylindrical bubble shape by replacing *F*_*s*_ with ${F}_{c}{n}_{{\rm{V}}}^{-1/3}$ and *γ* with *γ*/2. The correlation in Eq. 24 is very similar to (though not the same as) a plot of the right-hand side of the inequality in Eq. 23 with (2*γ* + *Gb*) replaced by $\sqrt{4{\gamma }^{2}+2\gamma Gb}$, which is the geometric mean of 2*γ* and (2*γ* + *Gb*). This results in the (more manageable) correlation

25 $$\begin{array}{cc}{n}_{{\rm{H}}{\rm{e}}} & ={n}_{{\rm{V}}}\Omega \,[{f}_{1}(T){F}_{s}^{1/3}{(4{\gamma }^{2}+2\gamma Gb)}^{-1/6}{n}_{{\rm{V}}}^{1/9}+{f}_{2}(T){F}_{s}^{2/3}{(4{\gamma }^{2}+2\gamma Gb)}^{-1/3}{n}_{{\rm{V}}}^{2/9}\\  & \,+{f}_{3}(T){F}_{s}{(4{\gamma }^{2}+2\gamma Gb)}^{-1/2}{n}_{{\rm{V}}}^{1/3}{]}^{-1}.\end{array}$$

Values of the parameters in these models, used to give the results (solid curves) of Figs. 1 and 2, are given in Table 1. For cylindrical bubbles, we anticipate that a reasonable estimate of a cylinder’s radius would be *r* = 3.5*a*_0_ (i.e., *R*_cyl_ = 3.5), based on dislocation loops emitted from bubbles as observed in our prior work^38^.

It should be noted that there were no pre-existing vacancies in the simulations, nor was there a source of vacancies other than the free surface. It should also be noted that the surface tension used here (see Table 1) is based on a tungsten–vacuum interface; a tungsten–helium interface may well have a different surface tension.

There are significant numbers of bubbles in Fig. 2 that fall below the equilibrium threshold established by Eq. 23. These can be explained by the fact that we have not screened bubbles that have partially ruptured and re-sealed again. Particularly at high fluence, this is common behaviour^20,41^, and there is some experimental evidence that helium exchanges with sub-surface bubbles in a manner consistent with such partial rupture/refill cycles^64^. The only screening we have done is to remove all bubbles closer than 5 nm to a free surface. This was done to remove surface effects, which would otherwise dominate the physics of bubble growth. This screening for depth removes some, but not all, of the partial-rupture cases, particularly for large bubbles.

In principle, the average bubble size should be that predicted by an accurate equation of state corresponding to the pressure at loop-punching. However, the loop-punching pressure is the *mean* pressure associated with loop-punching: the pressure in the bubble goes from above the threshold to below the threshold as the loop punches out^49^. With this in mind, it is really the *average* bubble size that we should be considering. The mean bubble density as a function of size averaged across the seven MD simulations discussed in our prior work^19,20^ is shown in Fig. 3 along with several models. It is even more obvious from this analysis that the correlation based on the MLB equation (Eq. 23) provides very good agreement with the MD data and is the best choice, and the geometric mean of the equilibrium and loop-punching models based on the MLB equation, as in either Eqs. 24 or 25, is an excellent match to the average bubble size.

The MLB equation is the most accurate among those tested, but it should be noted that the temperatures here are more than 600 K above the range over which the equation of state parameters were determined^53^. A more recently published equation of state^65^ was fit at temperatures up to 1000 K and pressures up to 45 GPa, but the equation is pressure-explicit and contains terms up to ${({n}_{{\rm{V}}}/{n}_{{\rm{He}}})}^{9}$; developing an analytical equation for *n*_He_ in terms of *n*_*V*_ or vice-versa from this model is consequently impossible. Accordingly, we recommend Eq. 23 for use in applications that require helium bubble density–size behaviour.

## Conclusions

Our examination of the ideal gas, truncated virial, and Benedict equations of state—which should be interpreted to be increasing in accuracy (and complexity) in that order—has suggested that bubbles inside tungsten do, to first approximation, obey Eq. 5. Given the nature of loop-punching and the corresponding pressure, we confirm that the bubble pressure is governed by the inequalities in Eq. 5. Assuming the equation of state of Benedict^52^ as parametrised by Mills, Liebenberg, and Bronson^53^ to be accurate for helium in this temperature range, we recommend using the geometric mean of the equilibrium and loop-punching models, Equation 24, or its close approximation in Eq. 25 as a model for the mean size of bubbles in coarse-grained models of helium transport in metals. At the particular temperature of 933 K, Eq. 24 becomes

26 $${n}_{{\rm{H}}{\rm{e}}}=\frac{16.08{n}_{{\rm{V}}}}{{\left[21.8150{n}_{{\rm{V}}}^{2/9}-5.7637{n}_{{\rm{V}}}^{1/3}+2.2631{n}_{{\rm{V}}}^{4/9}-0.1988{n}_{{\rm{V}}}^{5/9}+0.0260{n}_{{\rm{V}}}^{2/3}\right]}^{1/2}}{\rm{a}}{\rm{t}}\,933{\rm{K}},$$

where the numerator and denominator have both been multiplied by 10^30^ and had their units cancelled for convenience. It should be noted that if only the first term in the denominator is retained, this correlation reduces to ${n}_{{\rm{He}}}=3.4{n}_{{\rm{V}}}^{0.89}$, which is very similar to the empirical correlation in Eq. 1. When working at another temperature, a new correlation can be generated directly from Eqs. 24 or 25 using values from Table 1. For bubble sizes for which sufficient statistics are available from molecular dynamics simulations at 933 K, Eq. 26 is an extremely accurate predictor of average helium concentration (density) in high-pressure bubbles.

The fact that there are no adjustable parameters in the model contained in Eq. 24 suggests that, in principle, the expression could be extended to helium bubbles in other metals by substituting appropriate values of the atomic volume, surface tension, shear modulus, and so forth. Consequently, we would expect Eq. 24 to be broadly applicable and useful in coarse-grained models of gas transport in metals.
